# Persistent financial adversity and cognitive aging: a life course investigation

**DOI:** 10.1093/geroni/igag054

**Published:** 2026-07-23

**Authors:** Yiwen Liu, Jacques Wels, Sarah-Naomi James, Sarah E Keuss, Jane Maddock, Thomas D Parker, Jean Stafford, Jonathan M Schott, Marcus Richards, Praveetha Patalay

**Affiliations:** Unit for Lifelong Health and Ageing, Department of Population Science and Experimental Medicine, University College London, London, United Kingdom; Unit for Lifelong Health and Ageing, Department of Population Science and Experimental Medicine, University College London, London, United Kingdom; Health & Society Research Unit, Université libre de Bruxelles, Brussels, Belgium; Unit for Lifelong Health and Ageing, Department of Population Science and Experimental Medicine, University College London, London, United Kingdom; Dementia Research Centre, UCL Queen Square Institute of Neurology, London, United Kingdom; Unit for Lifelong Health and Ageing, Department of Population Science and Experimental Medicine, University College London, London, United Kingdom; Dementia Research Centre, UCL Queen Square Institute of Neurology, London, United Kingdom; Department of Brain Sciences, Faculty of Medicine, Imperial College London, London, United Kingdom; Advanced Care Research Centre (ACRC), University of Edinburgh, Edinburgh, United Kingdom; Dementia Research Centre, UCL Queen Square Institute of Neurology, London, United Kingdom; UK Dementia Research Institute, Faculty of Brain Sciences, University College London, London, United Kingdom; Unit for Lifelong Health and Ageing, Department of Population Science and Experimental Medicine, University College London, London, United Kingdom; Unit for Lifelong Health and Ageing, Department of Population Science and Experimental Medicine, University College London, London, United Kingdom; Centre for Longitudinal Studies, Social Research Institute, University College London, London, United Kingdom

**Keywords:** Cognition, Cognitive decline, Neuroimaging, Brain atrophy

## Abstract

**Background and Objectives:**

The long-term effect of persistent financial adversity on cognitive aging remains unclear. We examined how sustained financial hardship across adulthood associates with midlife cognition, cognitive decline, and later-life brain health and whether impacts vary by sex, childhood socioeconomic circumstances (SECs), and genetic risk.

**Research Design and Methods:**

Using data from the 1946 British birth cohort (*N* = 2,759) and its neuroimaging sub-study, Insight 46 (*N* = 356–468), we linked financial adversity (low household income, financial hardships between ages 26 and 53) with cognitive performance at age 53, cognitive decline from ages 53 to 69, and neuroimaging measures at ages 69–71. We tested moderating roles of sex, childhood SEC, and APOE-ɛ4.

**Results:**

Increased exposure to low household income and financial hardships was associated with lower processing speed (−0.07 [−0.13, −0.02] and −0.05 [−0.11, −0.00]) and verbal memory at age 53 (−0.16 [−0.21, −0.11] and −0.10 [−0.15, −0.05]). This was followed by slower verbal memory decline, attributable to lower baseline scores. Persistent low income was associated with greater ventricular volume (*b* = 4.67 ml [1.01, 8.32]). Stronger associations between financial adversity and brain atrophy were found for male participants, those with lower childhood SEC, and APOE-ɛ4 carriers who were consistently more vulnerable.

**Discussion and Implications:**

Persistent financial adversity impacts cognitive performance by midlife and later-life brain atrophy, with larger effects for men, those from disadvantaged childhoods, and individuals with greater genetic risk. Supporting financially vulnerable working-age adults could help prevent dementia in an aging population.

Innovation and Translational Significance: This research provides novel, life-course evidence by linking prospective, longitudinal data on financial adversity with cognitive and neuroimaging outcomes over seven decades. It innovates by demonstrating that the persistence of financial hardship, not just single episodes, is crucial for brain health, and by identifying key vulnerable subgroups (male, APOE-ɛ4 carriers). For science, it bridges socioeconomic exposure with biological mechanisms of aging, showing how adversity can exacerbate genetic risk. For policymakers, it offers a powerful, evidence-based argument that chronic poverty reduction is a critical strategy for dementia prevention, highlighting the long-term cognitive cost of financial adversity.

Dementia is one of the leading contributors to the global burden of disease (Alzheimer’s Association, 2015; Prince et al., 2015), and the rates are estimated to triple to 150 million over the next three decades (Patterson, 2018). There is a need to better understand how modifiable environmental exposures may be associated with cognitive aging (Rossor & Knapp, 2015). The association between childhood and adulthood socioeconomic circumstances (SECs) and cognitive functioning has been shown at different stages of the life course (Koster et al., 2005; Landy et al., 2017; Lyu & Burr, 2016; Migeot et al., 2022). Financial adversity—commonly defined as having a lack of money or resources to provide basic household necessities (Mirowsky & Ross, 2001)—is also associated with cognitive impairment and decline (Anstey et al., 2021; Kiely et al., 2019; Koster et al., 2005; Lee et al., 2010; Mani et al., 2013; Peterson et al., 2019).

In an Australian study using three waves of data collected between 2003 and 2015 where financial hardship was assessed using markers of scarcity (e.g., going on with basic needs, heat home) and behavioral response to hardship (e.g., pawning items or seeking help from the community), fixed effects modelling shows a longitudinal relationship between decline in cognitive abilities and markers of scarcity but not response to hardship (Kiely et al., 2019). Using cross-sectional U.S. data (2015–2016), another study clustering data by state shows that income inequality does not affect subjective cognitive decline, but a gradient is observed at the individual level when looking at individuals’ incomes (Peterson et al., 2019). Similarly, using cross-sectional data shows a strong association between low levels of education, low incomes, and few assets and cognitive decline using the Modified Mini-Mental State Examination score (Koster et al., 2005).

Using cross-sectional data and focusing on biomarkers of cognitive and memory decline by sex, it was evidenced that both childhood financial hardship and current financial problems are associated with lower cognitive scores among men. The relationship, however, is much smaller and not significant in women (Anstey et al., 2021). Such a sex difference contradicts previous findings on the association between accumulated financial strain and women’s self-rated health, where financial difficulties over the life course were linked to poorer self-reported health. This highlights the need to re-examine sex differences in cognitive aging when assessing the impact of financial adversity (Shippee et al., 2012).

Using the baseline wave of the Korean Longitudinal Study of Aging on respondents aged 65 and older, respondents’ cognitive status was assessed using the Mini-Mental State Examination and regressed on a risk score combining information on education attainment, income, wealth, and occupation. Results evidenced that each risk factor is associated with cognitive decline but that the combined effect of each factor is stronger than its separate effects (Lee et al., 2010). Other authors interested in understanding the causal nature of the relationship used both a laboratory study comparing rich and poor participants and a quasi-experimental field study established such a relationship, evidencing that, as the human cognitive system has limited capacity, budgetary preoccupations caused by financial adversity leave fewer cognitive resources available (Mani et al., 2013).

Although there is evidence for life course and intergenerational transmission of poverty (Harper et al., 2003; Moore, 2005), most studies to date use a snapshot measure of financial adversity or, in the best case, measures combining recent waves that do not capture the persistence of these experiences. One of the best attempts to address long-term accumulation was made using a within- and between-person approach using longitudinal data from the Health and Retirement Study, examining self-reported cognition between 1998 and 2010 (Lyu & Burr, 2016). It was observed that socioeconomic disadvantage in childhood (based on retrospective questions) is associated with lower cognitive functions at baseline (at age 65 or older) and follow-up. Although the study was based on childhood retrospective questions and did not follow respondents throughout their life course, it highlighted the necessity to address how financial hardship accumulates over time.

It has long been evidenced, for instance, through the Social Determinants of Health Framework (Wilkinson & Marmot, 1998), that there are broader social and economic contexts in which both financial hardship and insecurity occur. This framework suggests that socioeconomic inequality, which often manifests as financial hardship, increases the risk of poor mental health outcomes. Moreover, as economic insecurity increases, so does the prevalence of poor mental health, particularly in socioeconomically disadvantaged groups. These issues are compounded by structural factors such as unemployment, low wages, and poor working conditions, which all contribute to higher levels of financial insecurity and mental health difficulties. This is also true for cognitive decline. The present study argues that it is not only the recent exposure to financial adversity that should be considered but rather the long-term and accumulation of financial adversity throughout the life course that affects cognitive decline. Over time, the accumulation of such hardships can exacerbate health impairments (e.g., physical health, psychological distress, and cognition). For instance, the cumulative inequality theory argues that early-life disadvantages, including financial hardship, accumulate and magnify across the life course, producing a trajectory of disadvantage that results in poorer health outcomes in adulthood. This theory suggests that persistent economic challenges have a compounding effect, leading to negative health consequences over time (Ferraro & Shippee, 2009). There is evidence to suggest that a large proportion of the effect of childhood economic difficulty can be attributed to the continuity of financial adversity in adulthood, and that this accumulation of adversity showed stronger associations with health in later life (Luo & Waite, 2005). There is also evidence to suggest that financial adversity in adulthood has more implications for cognitive aging than exposures in childhood (Racine Maurice et al., 2021), and sustained exposure to financial adversity in adulthood has also been associated with worse cognitive functioning in aging (Foverskov et al., 2020; Zeki Al Hazzouri et al., 2017).

Life course analyses are interested in the “sequential unfolding of the outcomes of a person’s life” (Abbott, 2016). Individuals’ trajectories imply a continuity, and this translates into an accumulation of experiences, events, or exposures over time. At the center of the life course approach, the notion of accumulation is a key concept (Ben-Shlomo & Kuh, 2002; Kuh et al., 2003) that has gained prominence in research on aging, health, and social stratification but the term is not properly defined and, if defined, there are inconsistencies in its conceptualization (Ferraro & Morton, 2018). One of the earliest descriptions of the accumulation of risk was Riley’s concept of “insult accumulation” (Kuh et al., 2003) the notion that “life course exposures or insults gradually accumulate through episodes of illness and injury, adverse environmental conditions, and health damaging behaviours” (p. 779). Most life course concepts fall into three categories (Kuh et al., 2003): concepts referring to the causal pathway in relation to time (accumulation, chain of risk, and trajectory); concepts about the timing of causal actions (birth cohorts, critical and sensitive periods, induction, and latency periods); and concepts referring to different types of mechanisms (embodiment, mediating and modifying factors, resilience, susceptibility, and vulnerability). Patterns of the effects vary according to timing and duration of poverty exposure, and further longitudinal research is needed to disentangle time-specific from cumulative effects of poverty on child health (Béatrice et al., 2012).

Life course financial adversity has been assessed using either objective (e.g., household income) or subjective (e.g., perceived financial hardships such as inability to pay bills) indicators, with only one study identified that examined both in relation to cognitive function in midlife (Zeki Al Hazzouri et al., 2017). Although cognitive aging is often assessed as cognitive impairments or individual rates of decline using neuropsychological measures, neuroimaging studies can also index brain health markers that are associated with cognitive decline and dementia (Kloppenborg et al., 2014; Miquel et al., 2018; Prins & Scheltens, 2015; Ritchie et al., 2015). The association between persistent experiences of financial adversity and cognitive aging may also be moderated by other biological or environmental factors, including sex, genetic risk, or childhood SEC, which remains to be investigated. APOE-ε4 is the primary genetic risk factor for Alzheimer’s disease and is associated with increased cognitive decline in both clinical (Song et al., 2018) and nonclinical populations (Rusted & Carare, 2015). It has also been found to moderate the effect of other risk factors (e.g., neurodegenerative biomarkers, depression, childhood SEC) on cognitive functioning (Moorman et al., 2018; Ng et al., 2025; Piers et al., 2021), but its potential role in moderating the effect of persistent financial adversity has not yet been investigated.

Testing the longitudinal association between persistent financial adversity and cognitive aging requires the use of studies that have followed participants through multiple stages of development, such as the 1946 National Survey of Health and Development (NSHD) cohort. It is one of the oldest continuously running birth cohort studies with more than 70 years of follow-up since birth (Kuh et al., 2016), with repeated measures of financial adversity and cognitive function, and an embedded neuroimaging sub-study. Furthermore, extensive information collected in childhood allows controlling for potential confounder variables (particularly childhood cognitive ability) and enables testing of potential moderating effects of sex, genetic risk, and childhood SEC (Lu et al., 2019; Richards et al., 2019).

The overall aim of this study was to examine the effect of persistent financial adversity across adulthood on indicators of cognitive aging including cognitive decline and markers of brain health. We specifically investigated three questions: (a) What is the association between persistent financial adversity and cognitive function from mid-to-late adulthood? (b) What is the association between persistent financial adversity and markers of brain health? and (c) Is there a moderating effect of sex, childhood SEC, and genetic risk (APOE-ε4)?

By doing so, this study offers two key contributions to the understanding of the relationship between financial adversity and cognitive aging. First, it applies accumulation theories using prospective, longitudinal data rather than retrospective accounts, enabling a more robust examination of how financial hardship across both childhood and adulthood influences cognitive outcomes. Second, it extends beyond self-reported cognitive measures by incorporating objective neuroimaging markers of brain health, providing a more comprehensive and biologically grounded assessment of cognitive aging.

## Method

### Data

The MRC 1946 NSHD (*N* = 5,362) is a national British birth cohort of people born in one week of March 1946 (http://www.nshd.mrc.ac.uk/nshd) (Kuh et al., 2016). The main analytical sample included participants still alive at age 69 and with available cognitive assessments at ages 53, 63, or 69 (*N *= 2,759) (see Supplementary Figure 1). Ethical approval has been obtained from the Greater Manchester Local Research Ethics Committee and the Scotland Research Ethics Committee, and participants provided written informed consent at each data collection.

Participants recruited to the Insight 46 sub-study were identified from NSHD participants who had not previously withdrawn, died, or remained untraceable by the age of 69. Eligibility for recruitment included having life course data available and being able to attend a London-based clinic. Of the 841 participants who were invited, 502 [mean (*SD*) age 70.7 (0.7) years] attended the clinic for β-amyloid (Aβ) positron emission tomography (PET) and MR imaging based on previously published criteria (James et al., 2018; Lane et al., 2017). Of those who attended, 471 completed a brain scan at baseline, and 369 completed a follow-up scan [mean (*SD*) scan interval 2.4 (0.2) years] (Supplementary Figure 1).

### Measures

#### Financial adversity (ages 26–53)

##### Household income

Total household income was assessed at ages 26, 43, and 53 and was comparable to the population average at the time (see Supplementary Material for details). A binary variable was derived at each time point by grouping those with household income in the bottom 20% of the analytical sample (Morelli et al., 2021) into the low-income category. These were then summed across the three time points, providing a count of experiences of low household income (0 to 3) and further grouped into none (0), intermittent (1), or persistent experience (≥2).

##### Financial hardships

Financial hardships were assessed at ages 36, 43, and 53 (see the Supplementary Material). A binary variable was derived at each time point and summed across the three time points, providing a count of experiences of financial hardships (0 to 3). This was further grouped into none (0), intermittent (1), or persistent experience (≥2).

#### Cognitive function (at ages 53, 63, and 69)

Processing speed and verbal memory were assessed using the same assessments at ages 53, 63, and 69, with scores standardized across all ages. These assessments were first implemented in 1989 and have been consistently used to assess cognitive functioning in NSHD. They were chosen due to their sensitivity to age- and morbidity-related decline and can be easily administered according to a standardized protocol, have limited floor/ceiling effect, and are sensitive to change over time (Houx et al., 2002).

##### Processing speed

This was assessed using a timed-letter search task where participants had to cross out target letters “P” and “W” embedded among non-target letters within 1 min. A score represented the position reached at the end of the trial (max = 600).

##### Verbal memory

This was a word learning task, where participants were shown a list of 15 words over three administrations and were asked to write down as many as they could remember at the end of each. A score indicated the number of words correctly recalled across all administrations (max = 45).

#### Brain health

The full neuroimaging protocol for Insight 46 has been published (Lane et al., 2017). Structural MRI sequences first underwent correction for gradient non-linearity (Jovicich et al., 2006), brain-masked N4-bias correction (Tustison et al., 2010), and visual inspection of image quality. The following measures were included (detailed description is provided in Supplementary Material): (a) baseline (at ages 69–71) global white matter hyperintensity volume (WMHV); (b) baseline β-amyloid (Aβ) burden (standardized uptake value ratios [SUVRs]) and status (positive/negative); (c) baseline whole brain, ventricular and total hippocampal volume; and (d) longitudinal changes in whole brain, ventricular, and hippocampal volume between baseline and repeat MRI (71–74 years), calculated using the Boundary Shift Integral (BSI) (Freeborough & Fox, 1997; Leung et al., 2010a, 2010b) described elsewhere (Keuss et al., 2022).

#### Covariates

Covariates included: (a) sex at birth; (b) childhood SEC (paternal occupational status grouped into non-manual/skilled vs manual/unskilled/unemployed); (c) childhood cognition (8 years, summary measure of four tests from the National Foundation for Educational Research); (d) symptoms of depression and anxiety (13–15 years), rated by teachers and grouped into absent, mild or severe; and (e) educational attainment (26 years), grouped into ordinary (“O”)-level or below (equivalent to 11 years of education) versus advanced (“A”) level or above, with the latter as the reference. Total intracranial volume, calculated using Statistical Parametric Mapping 12 (Malone et al., 2015), was included as a covariate in models with volume outcomes, to adjust for head size (Lu et al., 2019).

#### Moderators

Sex, childhood SEC, and APOE-ε4, which was genotyped using two SNPs (rs439358 and rs7412) at age 53 and grouped into non-carrier vs carrier (homozygous or heterozygous), were used as moderators.

### Method

#### Cognitive function outcomes

A latent growth curve (LGC) model was used to examine changes in cognitive function over time (Duncan et al., 2006). Latent variables were generated for the intercept (baseline performance at age 53) and slope (change from age 53 to 69, with lower scores indicating greater decline). Time in the model was centered on the first assessment period (at age 53) and converted to decades. The statistical formula can be found in Supplementary Material. Full information maximum likelihood was used to estimate the cognition intercept and slope within the LGC model. After deriving the cognition intercept and slope from LGC models, linear regressions were estimated to examine their associations with persistent financial adversity (low household income and financial hardships, which were examined in separate models and treated as ordered variables to assess linear trends). These models were adjusted for all covariates. To test for non-linearity in dose–response, low household income and financial hardships were additionally treated as categorical variables in regression models, and quantile regression analysis investigated whether the effect of persistent financial adversity was similar across different quantiles (25th and 75th) of cognitive decline (see Supplementary Material). To examine effect modification, we included interaction terms between indicators of financial adversity and each of the modifiers on cognition intercept and slope in separate models. Effect modifications with *p*-value <.10 were visualized using predicted means from the model.

#### Brain health outcomes

The association between persistent financial adversity and markers of brain health was estimated using logistic regression (for Aβ status) and linear regression (all other outcomes). WMHV was log-transformed due to its positively skewed distribution. Longitudinal change in volumetric measures (BSI) was further adjusted for the time interval between MRI scans by dividing each BSI metric by the time (years) between scans (to represent change per year). Three models were fitted for each marker of brain health: the first examined the main effect of financial adversity, adjusted for sex and intracranial volume (for volumetric measures). The second examined the main effect of each moderator, adjusted for all covariates. The third examined the interaction between financial adversity and each moderator, adjusting for all covariates. Significant interactions were plotted using predicted means from the model.

All statistical analysis was performed in R (version 3.6.2).

### Missing data

To account for attrition, inverse probability weighting (see Supplementary Material) was used to calculate non-response weights for the analytic sample compared to the whole NSHD sample. Within the analytic sample, multivariable imputation by chained equations was used (20 imputations) for other study variables (see Supplementary Material) (Azur et al., 2011; Buuren & Groothuis-Oudshoorn, 2011).

### Additional and sensitivity analyses

Additional interactions were tested between the level of financial adversity (persistent or intermittent) associated with cognitive decline and each moderator, and between low household income and financial hardships (if both were associated with any outcome of interest). Four sensitivity analyses were also included to check the robustness of findings; primary analyses were repeated: (a) without sample weightings; (b) within Insight 46 participants only; (c) using a household size-adjusted low-income variable; and (d) performing multiple imputation prior to performing LGC analysis.

## Results

Of 2,759 participants included in the main analytical sample, 16% and 12% reported persistent low household income and financial hardships. The majority (73.6%) of those who never experienced low household income also never experienced financial hardships, and vice versa (66.9%) (Table 1).

**Table 1:** Sample characteristics.

Variables	Overall	Low household income			Financial hardships		
		None	Intermittent	Persistent	None	Intermittent	Persistent
***N***	2,759	1,574	733	426	1,727	689	313
**Low household income, *N* (%)**							
None	1,574 (57.6)	—	—	—	1,150 (66.9)	322 (47.0)	90 (28.8)
Intermittent	733 (26.8)	—	—	—	412 (24.0)	223 (32.6)	93 (29.7)
Persistent	426 (15.6)	—	—	—	156 (9.1)	140 (20.4)	130 (41.5)
**Financial hardships, *N* (%)**							
None	1,727 (63.3)	1,150 (73.6)	412 (56.6)	156 (36.6)	—	—	—
Intermittent	689 (25.2)	322 (20.6)	223 (30.6)	140 (32.9)	—	—	—
Persistent	313 (11.5)	90 (5.8)	93 (12.8)	130 (30.5)	—	—	—
**Sex: female, *N* (%)**	1,416 (51.3)	743 (47.2)	408 (55.7)	255 (59.9)	913 (52.9)	333 (48.3)	156 (49.8)
**Childhood socioeconomic circumstances: Unskilled/unemployed, *N* (%)**	241 (11.0)	104 (8.5)	75 (12.7)	58 (16.5)	135 (9.8)	61 (11.2)	44 (18.3)
**Childhood symptoms of depression and anxiety, *N* (%)**							
Absent	1,260 (51.2)	769 (55.1)	319 (48.6)	163 (41.3)	802 (51.5)	318 (53.0)	129 (45.3)
Mild	936 (38.0)	511 (36.6)	257 (39.2)	164 (41.5)	596 (38.3)	218 (36.3)	116 (40.7)
Severe	265 (10.8)	116 (8.3)	80 (12.2)	68 (17.2)	158 (10.2)	64 (10.7)	40 (14.0)
**Educational attainment: O-level or below, *N* (%)**	1,655 (63.2)	819 (54.7)	488 (70.4)	336 (81.8)	982 (59.4)	442 (68.5)	218 (73.9)
**APOE-ɛ4 status: carrier, *N* (%)**	702 (30.1)	413 (31.0)	186 (29.3)	102 (28.7)	452 (30.9)	173 (29.0)	77 (28.7)
**Processing speed, mean (*SD*)**							
At age 53	283.61 (75.35)	287.62 (73.55)	283.50 (76.09)	269.09 (78.41)	286.61 (74.35)	282.80 (77.66)	269.23 (74.19)
At age 63	267.12 (71.33)	271.39 (70.29)	266.79 (73.47)	249.46 (69.73)	268.05 (71.47)	268.31 (70.84)	255.76 (71.03)
At age 69	262.41 (74.18)	263.94 (70.54)	264.61 (80.39)	250.39 (75.96)	264.28 (73.49)	261.94 (75.60)	253.07 (73.95)
**Verbal memory, mean (*SD*)**							
At age 53	24.13 (6.25)	25.19 (6.01)	23.45 (6.42)	21.55 (5.86)	24.72 (6.14)	23.37 (6.26)	22.54 (6.37)
At age 63	24.28 (6.10)	25.15 (6.05)	23.72 (5.99)	21.54 (5.65)	24.83 (6.06)	23.46 (6.09)	22.84 (6.02)
At age 69	22.16 (6.07)	23.00 (5.97)	21.29 (6.02)	20.16 (5.87)	22.66 (5.92)	21.57 (6.06)	20.68 (6.55)

### Cognitive function outcomes

Increased experience of financial adversity showed dose–response associations with processing speed [low household income: SE = −0.13, 95% CI (−0.18, −0.08); financial hardships: −0.09 (−0.14, −0.04)] and verbal memory [low household income: −0.34 (−0.40, −0.29); financial hardships: −0.24 (−0.30, −0.17)] at age 53. These effect sizes were somewhat attenuated after adjusting for covariates (Supplementary Table 1).

**Figure 1:**
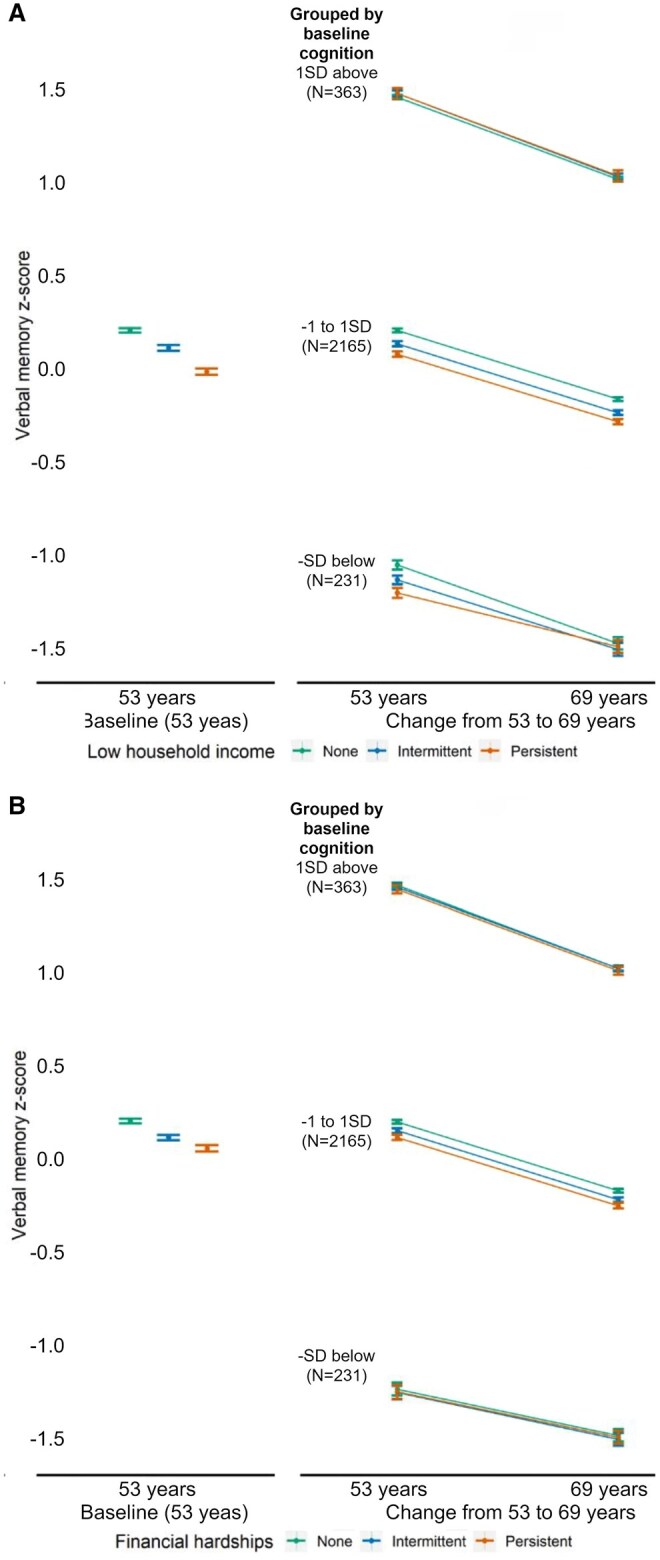
Predicted means on verbal memory at baseline (age 53) and decline (stratified on cognition at baseline) by financial adversity exposure. (A) Predicted means on verbal memory by low household income. (B) Predicted means on verbal memory by financial hardships.

There was overall weak evidence for an association between financial adversity and processing speed decline, but a small dose–response effect was found on verbal memory decline, with increased exposure associated with slower decline [low household income: 0.02 (0.01, 0.02); financial hardships: 0.01 (0.01, 0.01)] (Supplementary Table 1). Further testing of non-linearity showed that only persistent experience of financial adversity (rather than intermittent) was associated with slower verbal memory decline (Supplementary Tables 2 and 3). This may be explained by the fact that those with lower baseline cognitive performance also show a slower rate of decline over time. To illustrate this, we stratified the sample based on baseline cognition, and those scoring 1 *SD* or below at age 53 showed a slower rate of decline, particularly if they also persistently experienced financial adversity (Figure 1; Supplementary Figure 3).

**Figure 2:**
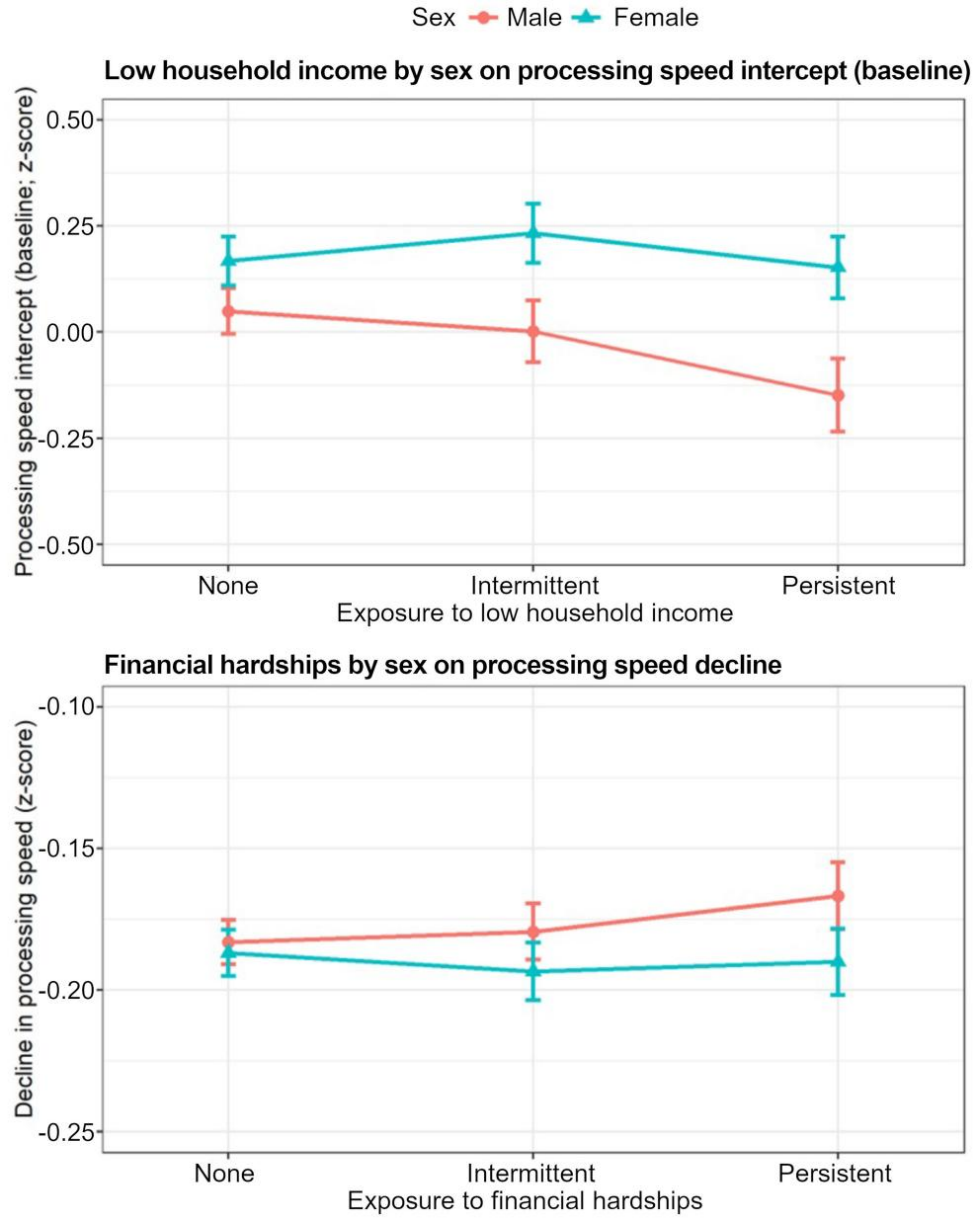
Predicted means on processing speed at baseline (age 53) and decline (lower scores representing faster decline) by financial adversity exposure and sex.

Sex moderated the effect of low household income on processing speed at baseline (age 53) (*p* = .009) and the effect of financial hardships on processing speed decline (*p* = .075; Supplementary Table 4). Post-hoc analyses showed that, compared to female participants, male participants who persistently experienced low household income scored lower on processing speed at age 53, but showed slower decline when persistently experiencing financial hardships (Figure 2). No other evidence of interactions was found for childhood SEC or genetic risk.

### Brain health outcomes

**Figure 3:**
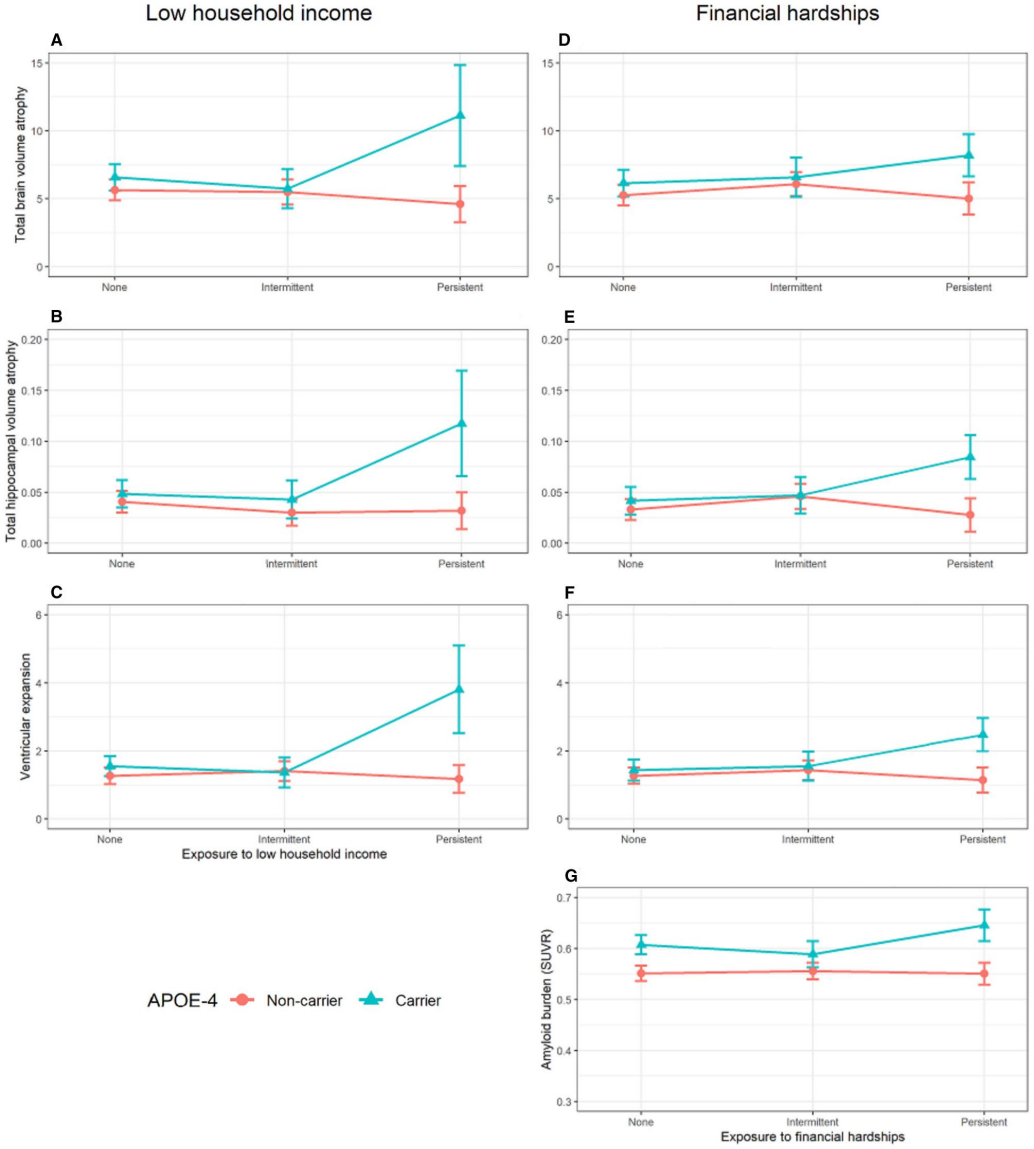
Predicted means on longitudinal measures of brain atrophy and baseline amyloid (Aβ) burden by financial adversity exposure and APOE-ɛ4 status. (A) Low household income × genetic risk on brain volume atrophy. (B) Low household income × genetic risk on hippocampal volume atrophy. (C) Low household income × genetic risk on ventricular expansion. (D) Financial hardships × genetic risk on brain volume atrophy. (E) Financial hardships × genetic risk on hippocampal volume atrophy. (F) Financial hardships × genetic risk on ventricular expansion. (G) Financial hardships × genetic risk on amyloid (SUVR).

**Figure 4:**
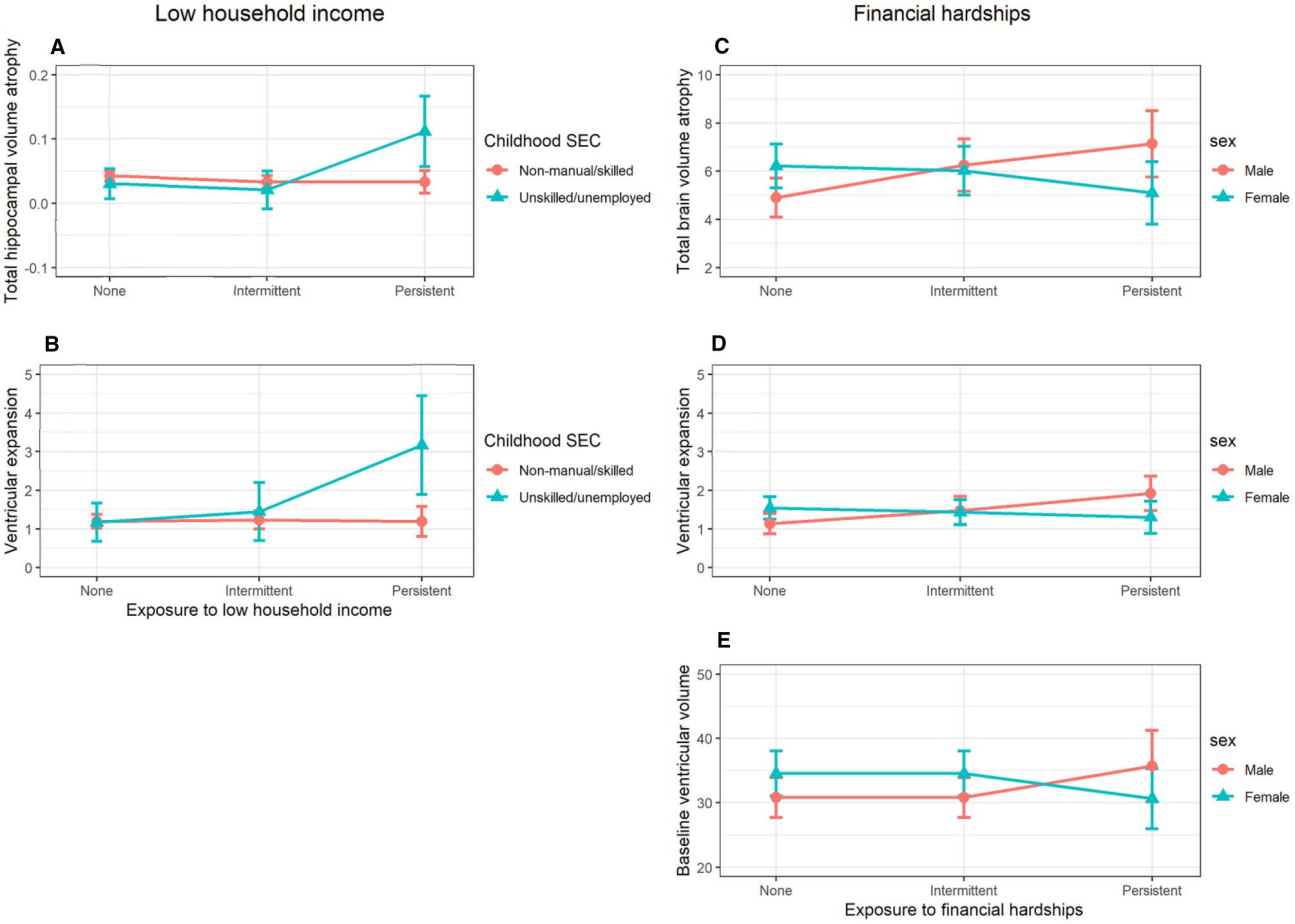
Predicted means on longitudinal measures of brain atrophy and baseline ventricular volume by financial adversity exposure and sociodemographic (sex, SEC) variables. (A) Low household income × childhood SEC on hippocampal volume atrophy. (B) Low household income × childhood SEC on ventricular expansion. (C) Financial hardships × sex on brain volume atrophy. (D) Financial hardships × sex on ventricular expansion. (E) Financial hardships × sex on baseline ventricular volume.

Increased low household income was associated with larger ventricular volume at ages 69–71 [*b* = 4.67 (1.01, 8.32)]. No other baseline associations were found (Supplementary Table 5). An interaction was found between financial hardships and APOE-ɛ4 on Aβ burden (SUVR) (*p* = .099), with carriers persistently experiencing financial hardships showing the highest Aβ burden (Figure 3G and Supplementary Table 5). An additional sex interaction was found with financial hardships on baseline ventricular volume (*p* = .046), with larger volume observed in male participants who persistently experienced financial hardships compared to female participants (Figure 4E and Supplementary Table 5).

No direct associations were found between financial adversity and longitudinal changes in volumetric measures. However, several interactions were identified (Supplementary Table 6). First, financial hardships and sex interacted on total brain volume atrophy (*p* = .003), and ventricular expansion (*p* = .005), with men who persistently experienced financial hardships showing faster brain atrophy and ventricular expansion compared to women (Figure 4C and D). Second, low household income and childhood SEC interacted on total hippocampal volume atrophy (*p* = .033) and ventricular expansion (*p* = .031), where those with disadvantaged childhood SEC who persistently experienced financial hardships across adulthood showed faster hippocampal atrophy and ventricular expansion compared to those with advantaged childhood SEC (Figure 4A and B). Finally, APOE-ɛ4 status showed both a main effect, as well as modified the effect of low household income and financial hardships on all volumetric changes (Supplementary Table 6). Carriers of APOE-ɛ4 who persistently experienced low household income or financial hardships showed faster total brain and hippocampal volume atrophy, as well as increased ventricular expansion, compared to non-carriers (Figure 3A–F).

### Additional and sensitivity analyses

No evidence of interactions was found for planned additional analyses (Supplementary Tables 7 and 8), and sensitivity analyses yielded similar effect sizes (outputs can be found in Supplementary Tables 9–15 and Supplementary Figures 4 and 5).

## Discussion

The aim of this study was to examine the effect of persistent financial adversity on cognitive aging. Findings add to the existing evidence base in at least four ways.

First, we investigated the persistence of both objective (low household income) and subjective (financial hardships) measures of financial adversity over many decades in the same individuals, and second, we focused on cognitive decline over almost two decades rather than a single measure at baseline. The findings that increased experience of both indicators of financial adversity was associated with lower scores on processing speed and verbal memory at age 53 support the accumulation life course model, but a slower rate of decline for verbal memory between ages 53 and 69 suggests substantial cognitive losses already present at midlife. One mechanism proposed for the role of financial adversity in cognitive impairment is that financial adversity may increase cognitive load, thus reducing the available cognitive bandwidth involved in processes such as attention and decision making (Mani et al., 2013; Sandi, 2013; Schilbach et al., 2016; Shah et al., 2012). Persistent experience of financial adversity in adulthood, therefore, may place chronic stress on systems involved in cognitive processing and lead to cognitive impairments over time. This effect persisted even after accounting for the effect of childhood SEC and supports previous findings that financial adversity in adulthood may have stronger associations with cognitive functioning in older age than adversity limited to childhood (Greenfield & Moorman, 2019; Racine Maurice et al., 2021). However, the finding that financial adversity was associated with a slower rate of decline is more reflective of early cognitive disadvantages rather than a protective effect of financial adversity and is similar to findings from another study, which showed faster cognitive decline among older adults with advantaged childhood SEC (Aartsen et al., 2019). This may be explained using the theory of cognitive reserve (Stern, 2009) that although advantaged financial situation is linked with higher mental stimulation and increased levels of cognitive reserve, there may be a time in older age when increased cognitive reserve can no longer compensate for neuronal loss, which may explain the accelerated decline among those with higher baseline function, as if they were “catching up.” Furthermore, the finding that only persistent exposure, rather than intermittent, was associated with cognitive decline offers additional support for the accumulation model and suggests that sustained financial adversity may be more detrimental to cognitive aging than any individual episode of adversity, although the latter was not specifically tested for in this study.

Third, we included neuroimaging data across two timepoints and found a dose-response association between low household income and increased ventricular volume at ages 69–71.

Fourth, we examined interactions with sex, childhood SEC, and APOE-ɛ4, which all moderated the effect of financial adversity on cognitive aging. APOE-ɛ4 in particular modified all associations with brain atrophy, such that carriers who persistently experienced low household income or financial hardships showed the highest rates of brain atrophy.

Sex also moderated the effect of financial adversity on processing speed, with men who persistently experienced financial adversity showing worse performance at age 53 (Camarata & Woodcock, 2006) and further showed a slower rate of decline over time compared to women, consistent with our proposal that those with lower cognitive performance at baseline show less decline over time. It has been suggested that men may be more vulnerable to the effects of financial adversity and low education on physical health outcomes (Halim et al., 2000; Odani et al., 2022), and findings here suggest that they may also be more vulnerable to cognitive outcomes, although the effect size was small.

A dose–response effect was also found between financial adversity and brain health, with increased experience of low household income associated with a 4.7 ml increase in ventricular volume at ages 69–71. In addition, there was evidence for multiple vulnerabilities to brain atrophy. Men appeared more vulnerable to the effect of persistent financial hardships on larger ventricular volumes and increased ventricular expansion, as well as increased total brain volume atrophy compared to women. Those with disadvantaged childhood SEC also appeared more vulnerable to the effect of persistent low household income on both hippocampal volume atrophy and ventricular expansion, extending previous findings (Butterworth et al., 2012; Elbejjani et al., 2017). APOE-ɛ4 carriers who were also persistently experiencing financial adversity showed higher rates of Aβ deposition (SUVRs), increased total brain and hippocampal atrophy, as well as ventricular expansion, highlighting a multiplicative effect where those with greater genetic risk are particularly vulnerable to the effects of persistent financial adversity, consistent with findings on the interaction between APOE-ɛ4 and education in other studies (Arenaza-Urquijo et al., 2015; Ferrari et al., 2013; Frank et al., 2021; Wang et al., 2012). One potential mechanism is that persistent adversity may reduce brain and cognitive reserve, which, when combined with genetic susceptibility to pathology, may render systems more vulnerable and decrease the threshold for clinical symptoms (Ferrari et al., 2013; Stern, 2009). Hence, the impact of persistent financial adversity on markers of brain health may be more complex depending on other co-existing factors such as sex, childhood SEC, and genetic risk, but the exploratory nature of the study, modest sample size, and issues with multiple testing will require further research to replicate and explain these findings and their mechanisms.

In this study, financial adversity can be conceptualized both as a direct psychosocial exposure—through mechanisms such as chronic stress and reduced cognitive bandwidth—and as a marker of broader cumulative socioeconomic disadvantage across adulthood. Although we adjusted for childhood SEC, financial adversity likely captures additional unmeasured structural and behavioral factors that accumulate over time. Therefore, while the findings are consistent with a potential causal role of persistent financial strain, financial adversity may also represent an indicator of sustained socioeconomic positioning and its associated exposures.

Some limitations of the study include selective attrition bias, with those who dropped out being more likely to have lower cognitive abilities and disadvantaged SEC (Kuh et al., 2016), particularly within Insight 46, which is a healthier and more socially advantaged cohort compared to the general population (James et al., 2018). This may lead to an underestimation of the effect of financial adversity on cognitive aging. There is also a lack of ethnic diversity in this cohort, and further research is needed using more ethnically representative populations.

Another limitation was the type of data collected for household income, which was a categorical variable indicating various income brackets rather than raw income values and not adjusted for household size; potentially reducing the precision of the low income variables derived (Overton et al., 2019). Pathways from financial adversity to markers of brain health were not investigated in this study, with one potential candidate being cerebrovascular dysfunction, which may precede neurodegenerative changes in the brain and cognitive decline (Kaufman et al., 2021; McCorkindale et al., 2022). Similarly, we did not examine major physical health conditions as part of this study, such as cardiovascular health, which is an important risk factor for cognitive impairment (Kanchva et al., 2026; Kulshreshtha et al., 2019) and dementia (Livingston et al., 2024), as well as being associated with financial stress (Swarup et al., 2024). All of these factors are plausible mechanisms in the causal pathway between financial adversity and cognitive outcomes. Further research investigating these additional pathways can shed more light on the consequences of persistent financial adversity and modifiable factors of dementia.

Finally, while the study focused on the accumulation of financial adversity over the life course, we did not examine the sources of incomes and, in particular, how unemployment and non-employment may affect brain health. Studies using the same cohort have shown detrimental associations of non-employment with cognitive aging (Sizer et al., 2025), and further research is needed to understand how the non-financial aspects of employment, including employment quality or underemployment (Vélez-Coto et al., 2021), influence cognitive aging.

## Conclusion

Persistent financial adversity is associated with reduced cognitive function in midlife and subsequently slower decline into older age, highlighting substantial cognitive losses already present in midlife. Men, those with disadvantaged childhood SEC and greater genetic risk (APOE-ɛ4), were also more susceptible to the effect of persistent financial adversity on brain atrophy. Given the current climate of cost-of-living crisis where a record number of households are reporting financial adversity (Broadbent et al., 2023), these findings further illustrate the importance of supporting vulnerable households to prevent financial adversity from becoming chronic, which could also help protect against age-related cognitive impairments and disorders such as dementia.

This study makes several important contributions to the literature on cognitive aging and financial adversity by adopting a life course perspective and leveraging both objective (low household income) and subjective (financial hardship) measures of adversity assessed prospectively over multiple decades. It provides strong evidence that persistent financial adversity is associated with lower cognitive performance already by midlife, particularly in domains such as processing speed and verbal memory, suggesting that cognitive deficits may emerge earlier than often assumed. By including neuroimaging data, the study further demonstrates that long-term financial adversity is linked to structural brain changes, including increased ventricular volume and brain atrophy, with evidence of dose–response relationships. A key contribution is the identification of differential effects by sex: men who experienced persistent financial adversity performed worse on cognitive tasks at age 53 and showed greater brain atrophy than women, indicating that men in this cohort may be more susceptible to the adverse cognitive effects of financial strain. However, one limitation is that these sex differences may partly reflect the social and economic context of the 1946 birth cohort, in which traditional gender roles and employment patterns could have amplified the impact of financial adversity on men; such patterns may differ in more recent cohorts where gender roles have shifted. Finally, the study highlights how financial adversity interacts with early-life disadvantage and genetic risk to exacerbate brain aging, providing support for theories of cumulative inequality and demonstrating that long-term socioeconomic conditions play a critical role in shaping cognitive health into later life.

## Supplementary Material

igag054_Supplementary_Data

## Data Availability

Data will be made available to researchers upon request to the NSHD Data Sharing Committee. For more information, see: http://www.nshd.mrc.ac.uk/data. The study was preregistered at the Open Science Framework (https://osf.io/6y9nv).
