# Supplementary material for: Persistent financial adversity and cognitive aging: a life course investigation
Source: Innov Aging. 2026 Jul 23;10(8):igag054. doi: 10.1093/geroni/igag054 (PMC13395473; doi:10.1093/geroni/igag054)
Supplement: igag054_Supplementary_Data [file igag054_supplementary_data.pdf]

***Innovation in Aging* Supplementary Material: Liu, Wels, James, Keuss, Maddock, Parker, Stafford, Schott, Richards, & Patalay. Persistent financial adversity and cognitive ageing: A life course investigation.**

Contents

Methods ... 2
Sample selection and characteristics ... 2
Measures ... 3
Analysis and statistical equations ... 4
Secondary analyses ... 6
Missing data ... 6
Sensitivity analyses ... 7
Deviation from pre-registration ... 7
Cognitive function outcomes ... 10
Brain health outcomes ... 15
Additional analyses ... 18
Sensitivity analyses ... 19
1. Repeat primary analyses without sample weighting ... 19
2. Repeat primary analyses in Insight46 sub-sample ... 20
3. Repeat primary analyses when low household income is derived within each family size grouping ... 22
4. Perform multiple imputation on cognitive scores and estimate cognitive decline and the role of financial adversity within SEM framework ... 24

Methods

Sample selection and characteristics

Sample selection for NSHD and Insight 46 can be found in Figure S1. Those who continued participation in NSHD were more likely to be in non-manual oc,cupations, have higher educational attainment and childhood cognitive ability compared to non-responders.[1] Participants recruited to Insight 46 were more likely to be from higher SEC, with better mental health and self-rated health, and were less likely to be obese or a current smoker compared to those who were invited but did not attend the clinic.[2]

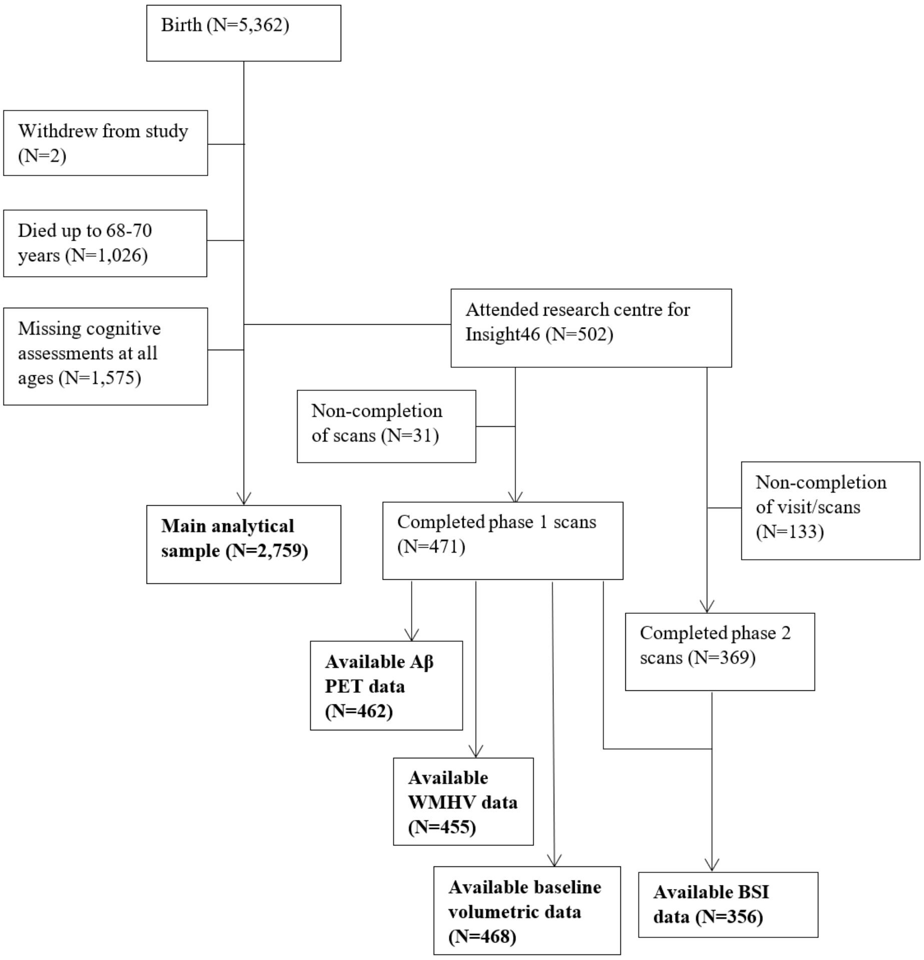

Figure S1. Sample flow chart.

Abbreviations: Aβ = β-amyloid; PET = positron emission tomography; WMHV: white matter hyperintensity volume; BSI = Boundary Shift Integral (assessing changes in brain volumes between baseline and follow-up).

Measures

Household income

Household income included regular payments as well as any benefits or pensions received, after deductions for tax and national insurance. This was reported as a monthly figure at age 26 and as an annual figure at 43 and 53 years. Participants were presented with cards indicating different income brackets and were asked to choose the card that best matched their own. Income brackets ranged from <£40 to ≥£200 monthly at 26 years (in £20 increments, i.e., £40-£59, £60-£79), and from <£2,000 to ≥£50,000 annually (in £1000 increments up to £12,000, followed by £12,000-£14,999, and in £5000 increments thereafter) at 43 and 53 years. The median household income in this cohort was £120-129 monthly at 26 years (UK in 1972: £133.6 for males, £74.4 for females), £11,000-£11,999 annually at 43 years (UK in 1989: £13,109), and £20,000-£24,999 annually at 53 years (UK in 1999-2000: £22,004).[3,4]

Financial hardships

Financial hardships were assessed at 36, 43, and 53 years. Across all three time points, participants were asked (i) how they managed on their current income (hard to manage, manage fairly well, or manage very well), and (ii) whether they had to go without things in the last 12 months due to a shortage of money (yes or no). An additional item on whether participants (iii) had trouble paying for bills in the last 12 months (never, sometimes, or often) was included at 43 and 53 years only.

Brain health

(1) Baseline white matter hyperintensity volume (WMHV): Generated by applying Bayesian Model Selection (BaMoS), an unsupervised automated algorithm [5] to T1 and FLAIR MRI. A global WMHV measure was generated, which included subcortical grey matter but not infratentorial regions.

(2) Baseline Aβ status and burden: Baseline Aβ PET data were acquired after intravenous injection of the 18F Aβ PET ligand florbetapir. Aβ PET data from the 10-minute period,

approximately 50 minutes post-injection, was processed including pseudo-CT attenuation correction.[6] Baseline Aβ global SUVRs (standardized uptake value ratios) were generated from a composite cortical region of interest,[7] and an eroded subcortical white matter reference region. Aβ status (positive/negative) was determined by applying gaussian mixture modelling to global SUVRs, and taking the 99th percentile of the lower gaussian as the cut-point for positivity (SUVR>0.6104).[8]

(3) Baseline whole brain, ventricular, and hippocampal volumes: Baseline whole brain and hippocampal volumes were automatically segmented from T1 MRI using Multi-Atlas Propagation and Segmentation (MAPS) and Similarity and Truth Estimation for Propagated Segmentation (STEPS), respectively.[9,10] Images were visually checked for segmentation errors and manually edited if needed. Baseline ventricular volumes were determined from T1 MRI using a semi-automated technique.[11]

(4) Longitudinal changes in whole brain, ventricular, and total hippocampal volume: Changes between baseline (age 69-71) and repeat T1 MRI (age 71-74) were calculated using the Boundary Shift Integral (BSI)[12–14] as described elsewhere.[15] All scan-pairs were reviewed to check that image quality was consistent between time points.

Analysis and statistical equations

Primary analyses

Change in cognitive functioning over time

Change in cognitive functioning between 53 and 69 years will be examined using latent growth curve (LGC) analysis. The formula for LGC is shown below:[16]

$$y_i = a_1 + t_i a_2 + \epsilon_i$$

Where y is the variable of interest (cognitive functioning) that changes with time i; a_1 and a_2 as the two latent variables representing the intercept and slope, respectively; t_i as the value of time; and ϵ_i as the error term (i.e., how much individuals vary from their predicted trajectory). Using the R (version 3.6.2) package 'lavaan', the following model can be specified for the intercept and slope within a SEM framework, where 'cog_1', 'cog_2' and 'cog_3' represent

cognitive scores at each time period of assessment; ‘i’ represents the latent variable ‘cognition intercept’, and ‘s’ represents the latent variable ‘cognition slope’ (Figure S2).

‘i =~ 1*cog_1 + 1*cog_2 + 1*cog_3

s =~ t_0*cog_1 + t_1*cog_2 + t_2*cog_3’

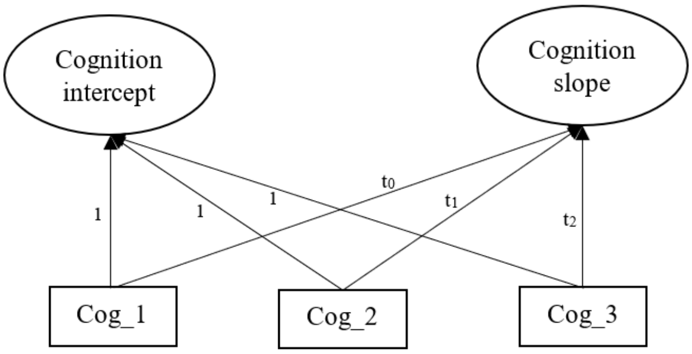

Figure S2. A LGC model on cognitive functioning across 3 time points.

Coefficients t_0, t_1, and t_2 in the model will be calculated using the below formulae, where ‘Age’ represents the actual age of assessment for each participant at each follow-up period, which is subtracted by age at first assessment and divided by 10 to represent time in decades since the first assessment. These are averaged across the analytical sample to calculate the mean follow-up period from the first assessment:

$$t_0 = \Sigma\left\{\frac{Age_{53} - Age_{53}}{10}\right\}/n$$

$$t_1 = \Sigma\left\{\frac{Age_{60-64} - Age_{53}}{10}\right\}/n$$

$$t_2 = \Sigma\left\{\frac{Age_{68-70} - Age_{53}}{10}\right\}/n$$

Persistent financial adversity predicting change in cognitive functioning over time

The impact of persistent financial adversity on cognition can be represented by the following formula, where y represents the latent variables cognition intercept and slope, and X represent the exposure variable (persistent financial adversity). This is then adjusted for the effect of covariates, denoted by C.

$$y = \beta_0 + \beta_1 X + \beta_2 \mathrm{C} + \epsilon$$

Effect modification model

The equation for the effect modification model is shown below, where X represents the exposure variable persistent financial adversity; M represents the moderator of interest (sex, childhood SES, or APOE-ε4); $X^{*}M$ as the interaction term between the exposure and moderator; and C as covariates in the model.

$$y = \beta_0 + \beta_1 X + \beta_2 M + \beta_3 X^{*} M + \beta_4 C + \epsilon$$

Secondary analyses

Quantile regression

The formula for a quantile regression model is shown below, where Q is the conditional quantile of the outcome y (i.e., cognitive decline), given a quantile level of τ which ranges between 0 and 1.

$$Q_\tau(y) = \beta_0(\tau) + \beta_1(\tau)X + \epsilon$$

Unlike linear regression, quantile regression offers insight into the effect of an exposure variable at different parts of the distribution of the outcome.[17] Quantile regression was performed at the 25th and 75th quantiles, representing those showing faster and slower decline, respectively.

Missing data

Missing data due to attrition

To reduce potential drop-out bias, we calculated non-response weights using Inverse Probability Weighting. Predictors for non-response in the NSHD cohort have been reported previously.[1,18] A multivariate regression model was used with these variables to predict the likelihood of non-response, which was inverted to calculate non-response weightings. Missing data on predictor variables of non-response were imputed using multivariable imputation by chained equations (MICE),[19,20] with 20 imputed datasets. Non-response weightings were pooled across the 20 datasets before merging with the analytical sample and used in subsequent analyses.

Missing data within the analytic sample

Auxiliary variables used for imputing missing data within the analytical sample included all variables in the analysis, along with other predictors of study variables, including personal income, occupational status, employment status, and marital status at 26, 36, 43, and 53 years. Imputation was performed on raw variables, with 20 imputed datasets generated, and any derived variables were calculated post imputation.

Sensitivity analyses

The following four sensitivity analyses were performed:

Steps from primary analyses were repeated:

(1) Without the inclusion of sample weightings to examine whether findings were substantially different.

(2) Within Insight 46 to investigate to what extent findings from the main sample replicate within this sub-sample.

(3) Third, to check whether the classification of low household income may be affected by household composition, we calculated the bottom 20% of the sample within each family size grouping (0 to 5+) to derive a household size-adjusted low income variable.

(4) Fourth, to check for potential implications of missing data strategies applied, we performed multiple imputation to handle missing data on cognitive function as well as all study variables prior to performing LGC analysis. The imputed datasets were then used to perform LGC analysis, first to estimate cognition intercept and slope, and second with added regression models to estimate the effect of financial adversity, with effects pooled across multiple imputed datasets.

Deviation from pre-registration

We initially only proposed to test for the interaction between financial adversity and sex, childhood SEC, and APOE-ε4 on cognition slopes. However, we also tested these interactions on cognition intercepts (baseline at 53 years), given the associations observed with baseline scores.

References

1. Kuh D, et al. The MRC National Survey of Health and Development reaches age 70: maintaining participation at older ages in a birth cohort study. *Eur J Epidemiol.* 2016;31(10):1135-1147. doi:10.1007/s10654-016-0217-8
2. James SN, et al. Using a birth cohort to study brain health and preclinical dementia: recruitment and participation rates in Insight 46. *BMC Res Notes.* 2018;11(1):1-9. doi:10.1186/s13104-018-3995-0
3. Office for National Statistics. Earnings timeseries of median gross weekly earnings from 1968 to 2021. Accessed April 16, 2026. https://www.ons.gov.uk/employmentandlabourmarket/peopleinwork/earningsandworkinghours/adhocs/13876earningstimeseriesofmediangrossweeklyearningsfrom1968to2021
4. Office for National Statistics. Average Incomes, Taxes and Benefits by Decile Groups of ALL Households. Accessed April 16, 2026. https://www.ons.gov.uk/peoplepopulationandcommunity/personalandhouseholdfinances/incomeandwealth/datasets/averageincomestaxesandbenefitsbydecilegroupsofallhouseholds
5. Sudre CH, et al. Bayesian model selection for pathological neuroimaging data applied to white matter lesion segmentation. *IEEE Trans Med Imaging.* 2015;34(10):2079-2102. doi:10.1109/TMI.2015.2419072
6. Burgos N, et al. Attenuation correction synthesis for hybrid PET-MR scanners: validation for brain study applications. *EJNMMI Phys.* 2014;1(suppl 1):A52. doi:10.1186/2197-7364-1-S1-A52
7. Landau SM, et al. Amyloid-β imaging with Pittsburgh Compound B and florbetapir: comparing radiotracers and quantification methods. *J Nucl Med.* 2013;54(1):70-77. doi:10.2967/jnumed.112.109009
8. Lu K, et al. Increased variability in reaction time is associated with amyloid beta pathology at age 70. *Alzheimers Dement (Amst).* 2020;12(1):e12076.
9. Leung KK, et al. Automated brain extraction using Multi-Atlas Propagation and Segmentation (MAPS). Paper presented at: 2011 IEEE International Symposium on Biomedical Imaging: From Nano to Macro; 2011:2053-2056. doi:10.1109/ISBI.2011.5872816

10. Cardoso MJ, et al. STEPS: Similarity and Truth Estimation for Propagated Segmentations and its application to hippocampal segmentation and brain parcelation. *Med Image Anal.* 2013;17(6):671-684. doi:10.1016/j.media.2013.02.006
11. Freeborough PA, Fox NC, Kitney RI. Interactive algorithms for the segmentation and quantitation of 3-D MRI brain scans. *Comput Methods Programs Biomed.* 1997;53(1):15-25. doi:10.1016/S0169-2607(97)01803-8
12. Freeborough PA, Fox NC. The boundary shift integral: an accurate and robust measure of cerebral volume changes from registered repeat MRI. *IEEE Trans Med Imaging.* 1997;16(5):623-629. doi:10.1109/42.640753
13. Leung KK, et al. Automated cross-sectional and longitudinal hippocampal volume measurement in mild cognitive impairment and Alzheimer's disease. *NeuroImage.* 2010;51(4):1345-1359. doi:10.1016/j.neuroimage.2010.03.018
14. Leung KK, et al. Robust atrophy rate measurement in Alzheimer's disease using multi-site serial MRI: tissue-specific intensity normalization and parameter selection. *NeuroImage.* 2010;50(2):516-523. doi:10.1016/j.neuroimage.2009.12.059
15. Keuss SE, et al. Associations of β-amyloid and vascular burden with rates of neurodegeneration in cognitively normal members of the 1946 British Birth Cohort. *Neurology.* 2022. doi:10.1212/WNL.0000000000200524
16. Duncan TE, Duncan SC, Strycker LA. *An Introduction to Latent Variable Growth Curve Modeling: Concepts, Issues, and Applications.* Routledge; 2006. doi:10.4324/9780203879962
17. Wei Y, Kehm RD, Goldberg M, Terry MB. Applications for quantile regression in epidemiology. *Curr Epidemiol Rep.* 2019;6(3):191-199. doi:10.1007/s40471-019-00204-6
18. Stafford M, et al. Using a birth cohort to study ageing: representativeness and response rates in the National Survey of Health and Development. *Eur J Ageing.* 2013;10(2):145-157. doi:10.1007/s10433-013-0258-8
19. Azur MJ, Stuart EA, Frangakis C, Leaf PJ. Multiple imputation by chained equations: what is it and how does it work? *Int J Methods Psychiatr Res.* 2011;20(1):40-49. doi:10.1002/mpr.329
20. van Buuren S, Groothuis-Oudshoorn K. MICE: multivariate imputation by chained equations in R. *J Stat Softw.* 2011;45(3):1-67. doi:10.18637/jss.v045.i03

Cognitive function outcomes

Table S1. Regression models showing the association between indicators of financial adversity (tested for linear trends) and cognition at baseline (intercept) and decline over time (slope).

	**Processing speed**						**Verbal memory**					
	**Decline (slope)**			**Decline (slope)**			**Baseline (intercept)**			**Decline (slope)**		
	**β**	**95%CI**	**p-value**	**β**	**95%CI**	**p-value**	**β**	**95%CI**	**p-value**	**β**	**95%CI**	**p-value**
**Low household income**												
Unadjusted	**-0.13**	**(-0.18, -0.08)**	**0.000**	**0.01**	**(0.00, 0.01)**	**0.025**	**-0.34**	**(-0.40, -0.29)**	**0.000**	**0.02**	**(0.01, 0.02)**	**0.000**
Adjusted	**-0.07**	**(-0.13, -0.02)**	**0.004**	0.01	(-0.00, 0.01)	0.099	**-0.16**	**(-0.21, -0.11)**	**0.000**	**0.01**	**(0.00, 0.01)**	**0.000**
**Financial hardships**												
Unadjusted	**-0.09**	**(-0.14, -0.04)**	**0.001**	0.01	(-0.00, 0.01)	0.099	**-0.24**	**(-0.30, -0.17)**	**0.000**	**0.01**	**(0.01, 0.01)**	**0.000**
Adjusted	**-0.05**	**(-0.11, -0.00)**	**0.040**	0.00	(-0.00, 0.01)	0.219	**-0.10**	**(-0.15, -0.05)**	**0.000**	**0.00**	**(0.00, 0.01)**	**0.023**

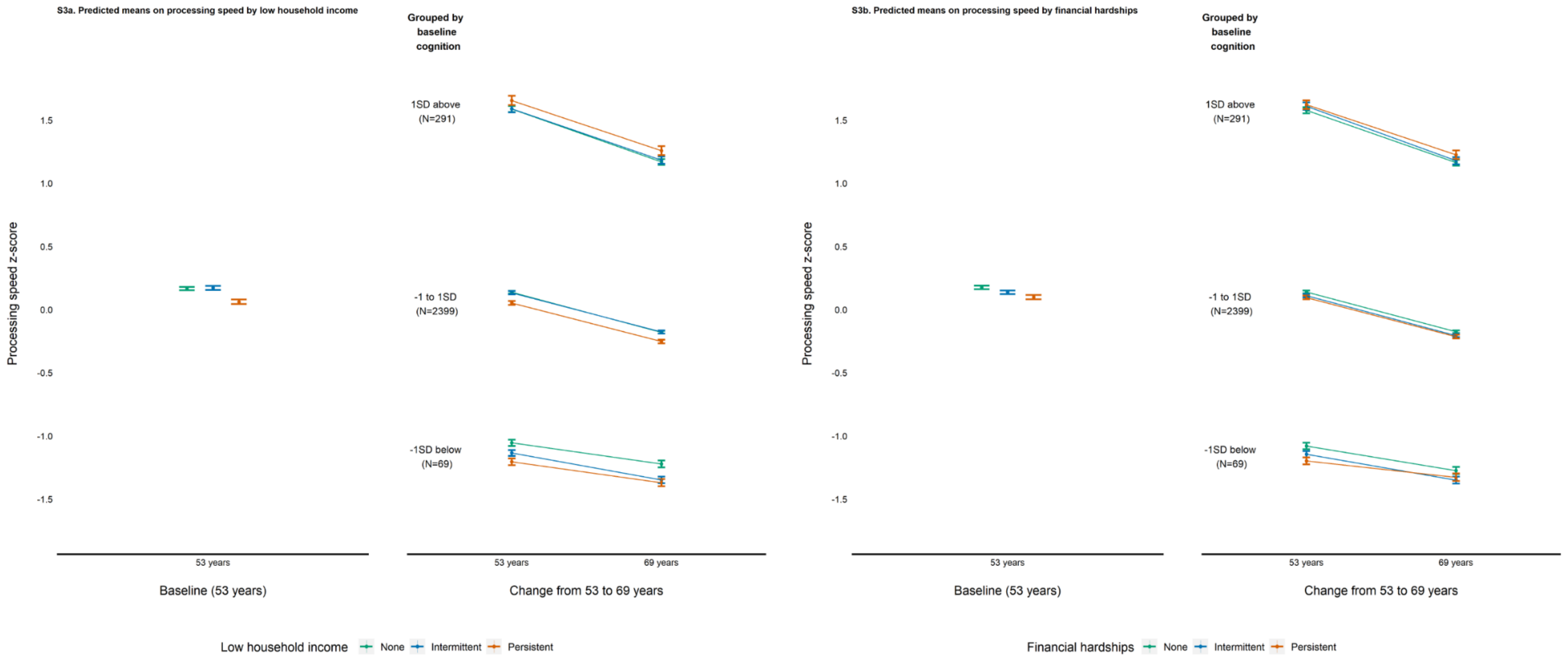

Figure S3. Predicted means on processing speed at baseline (53 years) and decline (stratified on cognition at baseline) by financial adversity exposure.

Table S2. Regression models showing the association between indicators of financial adversity (coded as categorical variables) and cognitive decline.

	Processing speed (decline)			Verbal memory (decline)		
	**b**	**95%CI**	**p.value**	**b**	**95%CI**	**p.value**
**Low household income**						
Never [Reference]						
Intermittent	0.00	(-0.01, 0.01)	0.569	0.00	(-0.00, 0.01)	0.539
Persistent	0.01	(-0.00, 0.02)	0.099	**0.01**	**(0.00, 0.01)**	**0.000**
**Financial hardships**						
Never [Reference]						
Intermittent	-0.00	(-0.01, 0.01)	0.831	0.00	(-0.00, 0.01)	0.364
Persistent	0.01	(-0.00, 0.02)	0.219	**0.01**	**(0.00, 0.01)**	**0.023**

All models adjusted for covariates.

Table S3. Regression models showing the association between indicators of financial adversity (as categorical variables) and cognitive decline at different quantiles, adjusted for all covariates.

	**Processing speed (decline)**			**Verbal memory (decline)**		
	**b**	**95%CI**	**p.value**	**b**	**95%CI**	**p.value**
**Faster decline [25th quantile]**						
Low household income [Intermittent]	0.00	(-0.01, 0.02)	0.436	-0.00	(-0.01, 0.01)	0.944
Low household income [Persistent]	**0.02**	**(0.00, 0.03)**	**0.014**	**0.01**	**(0.00, 0.02)**	**0.002**
Financial hardships [Intermittent]	0.00	(-0.01, 0.01)	0.664	0.00	(-0.01, 0.01)	0.624
Financial hardships [Persistent]	0.01	(-0.01, 0.02)	0.251	**0.01**	**(0.00, 0.02)**	**0.014**
**Slower decline [75th quantile**]						
Low household income [Intermittent]	0.01	(-0.01, 0.02)	0.271	0.00	(-0.00, 0.01)	0.247
Low household income [Persistent]	0.00	(-0.01, 0.01)	0.686	**0.01**	**(0.00, 0.02)**	**0.020**
Financial hardships [Intermittent]	0.00	(-0.01, 0.01)	0.953	0.00	(-0.00, 0.01)	0.390
Financial hardships [Persistent]	0.00	(-0.01, 0.02)	0.564	0.01	(-0.00, 0.01)	0.211

When quantile regression was performed at the 25th and 75th quantile (faster and slower decline, respectively), only persistent experience of low household income was associated with verbal memory at both quantiles, and additionally associated with processing speed at the 25th quantile only (faster decline) (Supplementary Table S3).

Table S4. Regression models showing the interaction between indicators of financial adversity and sex, childhood SEC and APOE-4 on cognition at baseline (intercept) and decline (slope).

	**Processing speed**						**Verbal memory**					
	**Baseline (intercept)**			**Decline (slope)**			**Baseline (intercept)**			**Decline (slope)**		
	**b**	**95%CI**	**p.value**	**b**	**95%CI**	**p.value**	**b**	**95%CI**	**p.value**	**b**	**95%CI**	**p.value**
**Low household income***												
Sex	**0.13**	**(0.03, 0.23)**	**0.009**	-0.00	(-0.02, 0.01)	0.721	-0.04	(-0.13, 0.05)	0.402	0.00	(-0.00, 0.01)	0.525
Childhood SEC	-0.08	(-0.24, 0.07)	0.308	0.01	(-0.01, 0.03)	0.430	-0.02	(-0.16, 0.13)	0.810	0.00	(-0.01, 0.01)	0.515
APOE-4	0.07	(-0.05, 0.19)	0.245	0.00	(-0.01, 0.02)	0.638	-0.02	(-0.13, 0.09)	0.732	-0.00	(-0.01, 0.01)	0.929
**Financial hardships***												
Sex	0.06	(-0.04, 0.16)	0.260	**-0.01**	**(-0.03, 0.00)**	**0.075**	0.00	(-0.10, 0.10)	0.994	0.01	(-0.00, 0.01)	0.131
Childhood SEC	-0.08	(-0.23, 0.07)	0.294	0.01	(-0.01, 0.03)	0.283	-0.08	(-0.23, 0.06)	0.259	0.00	(-0.01, 0.01)	0.833
APOE-4	0.00	(-0.12, 0.13)	0.945	-0.01	(-0.03, 0.01)	0.258	0.03	(-0.09, 0.15)	0.630	-0.01	(-0.01, 0.00)	0.213

All models adjusted for covariates.

Brain health outcomes

Table S5. Regression models showing the association between indicators of financial adversity (tested for linear trends) and β-amyloid (Aβ) and baseline volumetric measures.

	**Main effect of financial adversity**[a]			**Main effect of moderators**[b]			**Interactions**[b]		
	**OR**	**95%CI**	**p.value**	**OR**	**95%CI**	**p.value**	**OR**	**95%CI**	**p.value**
	**Aβ status**								
**Low household income**	0.02	(-0.69, 0.73)	0.960						
**Sex**				-0.03	(-0.59, 0.53)	0.909	-0.44	(-1.89, 1.02)	0.556
**Child SEC**				0.74	(-0.33, 1.81)	0.175	0.70	(-1.27, 2.66)	0.485
**APOE-ε4**				**1.65**	**(1.09, 2.22)**	**<0.001**	-0.16	(-1.87, 1.54)	0.850
**Financial hardships**	0.15	(-0.37, 0.68)	0.566						
**Sex**				0.04	(-0.53, 0.61)	0.889	0.39	(-0.71, 1.49)	0.486
**Child SEC**				0.72	(-0.33, 1.77)	0.176	-0.57	(-2.29, 1.15)	0.516
**APOE-ε4**				**1.58**	**(1.02, 2.13)**	**<0.001**	0.38	(-0.79, 1.55)	0.522
	**B**	**95%CI**	**p.value**	**B**	**95%CI**	**p.value**	**B**	**95%CI**	**p.value**
	**Aβ burden (SUVR)**								
**Low household income**	0.00	(-0.01, 0.02)	0.602						
**Sex**				-0.00	(-0.02, 0.01)	0.726	-0.02	(-0.06, 0.02)	0.313
**Child SEC**				0.01	(-0.02, 0.04)	0.349	0.03	(-0.03, 0.10)	0.337
**APOE-ε4**				**0.06**	**(0.04, 0.07)**	**<0.001**	0.01	(-0.04, 0.06)	0.764
**Financial hardships**	0.01	(-0.01, 0.02)	0.401						
**Sex**				-0.00	(-0.02, 0.01)	0.790	0.02	(-0.01, 0.05)	0.170
**Child SEC**				0.01	(-0.01, 0.04)	0.314	0.02	(-0.05, 0.08)	0.606
**APOE-ε4**				**0.05**	**(0.04, 0.07)**	**<0.001**	**0.03**	**(-0.01, 0.06)**	**0.099**
	**WMHV**[c]								
**Low household income**	0.01	(-0.24, 0.27)	0.918						
**Sex**				**0.35**	**(0.10, 0.60)**	**0.007**	0.07	(-0.45, 0.58)	0.804
**Child SEC**				0.18	(-0.24, 0.59)	0.406	-0.08	(-0.86, 0.71)	0.851
**APOE-ε4**				0.11	(-0.11, 0.33)	0.332	-0.06	(-0.84, 0.72)	0.874
**Financial hardships**	0.08	(-0.13, 0.29)	0.437						
**Sex**				**0.31**	**(0.06, 0.57)**	**0.016**	0.07	(-0.36, 0.50)	0.745
**Child SEC**				0.16	(-0.25, 0.57)	0.445	-0.24	(-1.04, 0.57)	0.563
**APOE-ε4**				0.12	(-0.11, 0.34)	0.307	0.03	(-0.44, 0.49)	0.911

	Main effect of financial adversity[a]			Main effect of moderators[b]			Interactions[b]		
	**Baseline total brain volume**								
**Low household income**	-4.20	(-15.25, 6.89)	0.455						
**Sex**				**22.59**	**(11.45, 33.73)**	**<0.001**	5.34	(-17.12, 27.80)	0.640
**Child SEC**				5.14	(-11.71, 21.99)	0.549	8.65	(-24.71, 42.00)	0.610
**APOE-ε4**				**9.91**	**(0.14, 19.68)**	**0.047**	-16.74	(-45.43, 11.94)	0.252
**Financial hardships**	6.79	(-2.36, 15.95)	0.146						
**Sex**				**19.35**	**(8.22, 30.47)**	**0.001**	10.80	(-7.91, 29.51)	0.257
**Child SEC**				3.51	(-12.99, 20.01)	0.676	-16.13	(-51.56, 19.30)	0.370
**APOE-ε4**				**10.58**	**(1.01, 20.16)**	**0.030**	-2.61	(-22.62, 17.39)	0.797
	**Baseline hippocampal volume**								
**Low household income**	-0.01	(-0.16, 0.14)	0.894						
**Sex**				-0.11	(-0.26, 0.04)	0.136	0.18	(-0.14, 0.50)	0.272
**Child SEC**				-0.17	(-0.41, 0.06)	0.137	-0.07	(-0.56, 0.42)	0.766
**APOE-ε4**				-0.06	(-0.19, 0.06)	0.316	0.12	(-0.29, 0.53)	0.559
**Financial hardships**	0.07	(-0.05, 0.19)	0.270						
**Sex**				-0.14	(-0.28, 0.01)	0.067	0.11	(-0.14, 0.37)	0.372
**Child SEC**				-0.19	(-0.41, 0.04)	0.108	-0.02	(-0.47, 0.44)	0.943
**APOE-ε4**				-0.06	(-0.19, 0.06)	0.337	-0.06	(-0.34, 0.21)	0.648
	**Baseline ventricular volume**								
**Low household income**	**4.67**	**(1.01, 8.32)**	**0.013**						
**Sex**				1.14	(-2.41, 4.68)	0.530	-5.29	(-12.60, 2.02)	0.155
**Child SEC**				-1.40	(-6.68, 3.89)	0.604	4.82	(-6.64, 16.28)	0.408
**APOE-ε4**				-1.43	(-4.52, 1.66)	0.363	3.72	(-6.08, 13.53)	0.455
**Financial hardships**	0.10	(-2.90, 3.10)	0.946						
**Sex**				1.77	(-1.85, 5.39)	0.338	**-6.20**	**(-12.30, -0.10)**	**0.046**
**Child SEC**				-7.22	(-6.08, 4.63)	0.791	0.13	(-11.25, 11.51)	0.982
**APOE-ε4**				-1.72	(-4.7, 1.32)	0.266	3.04	(-3.67, 9.74)	0.373

[a]Adjusted for sex and intracranial volume (for WMHV and baseline volumetric measures)
[b]Adjusted for all covariates
[c]Log-transformed

Table S6. Regression models showing the association between indicators of financial adversity (tested for linear trends) and longitudinal changes in brain health (per year over 2.4 years).

	Main effect of financial adversity[a]			Main effect of moderators[b]			Interactions[b]		
	**B**	**95%CI**	**p.value**	**B**	**95%CI**	**p.value**	**B**	**95%CI**	**p.value**
	**Total brain volume atrophy**								
**Low household income**	-0.20	(-1.19, 0.79)	0.696						
**Sex**				0.59	(-0.36, 1.54)	0.220	-0.70	(-2.68, 1.27)	0.483
**Child SEC**				0.00	(-1.45, 1.45)	0.998	1.58	(-2.55, 5.71)	0.446
**APOE-ε4**				**1.06**	**(0.20, 1.93)**	**0.016**	**3.96**	**(0.53, 7.39)**	**0.025**
**Financial hardships**	0.42	(-0.38, 1.22)	0.302						
**Sex**				0.41	(-0.54, 1.36)	0.398	**-2.37**	**(-3.95, -0.80)**	**0.003**
**Child SEC**				-0.04	(-1.50, 1.41)	0.953	1.40	(-2.48, 5.29)	0.473
**APOE-ε4**				**1.10**	**(0.24, 1.95)**	**0.012**	**1.63**	**(-0.06, 3.31)**	**0.058**
	**Total hippocampal volume atrophy**								
**Low household income**	0.00	(-0.01, 0.01)	0.908						
**Sex**				0.01	(-0.01, 0.02)	0.376	-0.01	(-0.04, 0.02)	0.506
**Child SEC**				0.00	(-0.02, 0.02)	0.852	**0.06**	**(0.01, 0.12)**	**0.033**
**APOE-ε4**				**0.01**	**(0.00, 0.02)**	**0.024**	**0.06**	**(0.01, 0.10)**	**0.028**
**Financial hardships**	0.01	(-0.00, 0.02)	0.169						
**Sex**				0.00	(-0.01, 0.02)	0.699	-0.01	(-0.03, 0.01)	0.524
**Child SEC**				0.00	(-0.02, 0.02)	0.871	0.04	(-0.02, 0.11)	0.197
**APOE-ε4**				**0.01**	**(0.00, 0.02)**	**0.021**	**0.03**	**(0.01, 0.06)**	**0.004**
	**Ventricular expansion**								
**Low household income**	0.21	(-0.10, 0.52)	0.188						
**Sex**				0.18	(-0.13, 0.48)	0.256	-0.09	(-0.72, 0.53)	0.767
**Child SEC**				0.36	(-0.09, 0.81)	0.120	**1.42**	**(0.14, 2.69)**	**0.031**
**APOE-ε4**				**0.33**	**(0.07, 0.59)**	**0.013**	**1.66**	**(0.51, 2.82)**	**0.006**
**Financial hardships**	0.21	(-0.04, 0.46)	0.105						
**Sex**				0.14	(-0.17, 0.45)	0.379	**-0.73**	**(-1.24, -0.22)**	**0.005**
**Child SEC**				0.37	(-0.08, 0.82)	0.111	1.34	(-0.38, 3.06)	0.122
**APOE-ε4**				**0.32**	**(0.06, 0.58)**	**0.017**	**0.83**	**(0.30, 1.36)**	**0.002**

[a]Adjusted for sex and intracranial volume
[b]Adjusted for all covariates

Additional analyses

We additionally tested for an interaction between persistent experience of financial adversity and sex, childhood SEC and APOE-ε4 on verbal memory decline only, given that only persistent experience, rather than intermittent experience, was associated with verbal memory decline from secondary analyses (Supplementary Table S2). No evidence of interactions was found (Supplementary Table S6). Furthermore, since both low household income and financial hardships were also associated with processing speed (baseline) and verbal memory (baseline and decline) from primary analyses, we further included an interaction term between them, and no interactions were found (Supplementary Table S7).

Table S7. Regression models showing the interaction between indicators of financial adversity and sex, childhood SEC and APOE-4 in relation to verbal memory decline.

	**Persistent low household income**			**Persistent financial hardships**		
**Verbal memory decline**	**b**	**95%CI**	**p.value**	**b**	**95%CI**	**p.value**
**Interaction with**						
Sex	0.00	(-0.00, 0.01)	0.325	0.01	(-0.00, 0.02)	0.175
Childhood SEC	0.00	(-0.01, 0.02)	0.828	0.00	(-0.01, 0.01)	0.980
APOE-4	-0.00	(-0.01, 0.01)	0.941	-0.01	(-0.02, 0.00)	0.153

Table S8. Regression models showing the interaction between low household income and financial hardships on verbal memory.

	**b**	**95%CI**	**p.value**
**Low household income x Financial hardships**			
Processing speed (intercept)	-0.08	(-0.17, 0.02)	0.113
Verbal memory (intercept)	-0.00	(-0.10, 0.09)	0.951
Verbal memory (decline)	0.00	(-0.01, 0.01)	0.811

Sensitivity analyses

1. Repeat primary analyses without sample weighting

Table S9. Regression models showing the association between indicators of financial adversity (tested for linear trends) and cognition at endpoint (intercept) and decline over time (slope), without sample weights.

	**Processing speed**						**Verbal memory**					
	**Endpoint (intercept)**			**Decline (slope)**			**Endpoint (intercept)**			**Decline (slope)**		
	**b**	**95%CI**	**p.value**	**b**	**95%CI**	**p.value**	**b**	**95%CI**	**p.value**	**b**	**95%CI**	**p.value**
**Low household income**												
Unadjusted	**-0.13**	**(-0.18, -0.08)**	**0.000**	**0.01**	**(0.00, 0.02)**	**0.033**	**-0.34**	**(-0.40, -0.29)**	**0.000**	**0.01**	**(0.01, 0.01)**	**0.000**
Adjusted	**-0.07**	**(-0.13, -0.02)**	**0.006**	0.01	(-0.00, 0.01)	0.095	**-0.16**	**(-0.21, -0.11)**	**0.000**	**0.00**	**(0.00, 0.01)**	**0.000**
**Financial hardships**												
Unadjusted	**-0.08**	**(-0.14, -0.02)**	**0.011**	0.00	(-0.00, 0.01)	0.237	**-0.21**	**(-0.27, -0.15)**	**0.000**	**0.01**	**(0.00, 0.01)**	**0.000**
Adjusted	-0.04	(-0.10, 0.02)	0.162	0.00	(-0.00, 0.01)	0.396	**-0.09**	**(-0.14, -0.04)**	**0.000**	**0.00**	**(0.00, 0.00)**	**0.024**

Table S10. Regression models showing the interaction between indicators of financial adversity and sex, childhood SEC and APOE-4 in relation to cognitive decline, adjusted for all covariates, without sample weights.

	**Processing speed (decline)**			**Verbal memory (decline)**		
	**b**	**95%CI**	**p.value**	**b**	**95%CI**	**p.value**
**Low household income***						
Sex	-0.00	(-0.01, 0.01)	0.965	0.00	(-0.00, 0.00)	0.690
Childhood SEC	0.01	(-0.01, 0.03)	0.308	0.00	(-0.00, 0.01)	0.529
APOE-4	0.00	(-0.01, 0.02)	0.611	0.00	(-0.00, 0.00)	0.911
**Financial hardships***						
Sex	-0.01	(-0.03, 0.00)	0.165	0.00	(-0.00, 0.01)	0.425
Childhood SEC	0.01	(-0.01, 0.04)	0.311	0.00	(-0.00, 0.01)	0.606
APOE-4	-0.01	(-0.03, 0.01)	0.356	-0.00	(-0.01, 0.00)	0.293

2. Repeat primary analyses in Insight46 sub-sample

Table S11. Regression models showing the association between indicators of financial adversity (tested for linear trends) and cognition at endpoint (intercept) and decline over time (slope), in Insight46 sub-sample only.

	Processing speed						Verbal memory					
	Endpoint (intercept)			Decline (slope)			Endpoint (intercept)			Decline (slope)		
	b	95%CI	p.value	b	95%CI	p.value	b	95%CI	p.value	b	95%CI	p.value
**Low household income**												
Unadjusted	-0.06	(-0.21, 0.09)	0.422	0.02	(-0.00, 0.05)	0.104	**-0.29**	**(-0.46, -0.12)**	**0.001**	**0.02**	**(0.00, 0.03)**	**0.017**
Adjusted	-0.05	(-0.21, 0.10)	0.516	0.02	(-0.01, 0.05)	0.126	**-0.25**	**(-0.40, -0.10)**	**0.001**	**0.01**	**(0.00, 0.03)**	**0.026**
**Financial hardships**												
Unadjusted	-0.05	(-0.17, 0.08)	0.447	0.01	(-0.01, 0.03)	0.242	-0.03	(-0.18, 0.11)	0.647	-0.00	(-0.01, 0.01)	0.414
Adjusted	-0.06	(-0.19, 0.06)	0.331	0.01	(-0.01, 0.03)	0.231	-0.06	(-0.18, 0.07)	0.378	-0.00	(-0.01, 0.01)	0.483

Table S12. Regression models showing the interaction between indicators of financial adversity and sex, childhood SEC and APOE-4 in relation to cognitive decline, adjusted for all covariates, in Insight46 sub-sample only.

	**Processing speed (decline)**			**Verbal memory (decline)**		
	**b**	**95%CI**	**p.value**	**b**	**95%CI**	**p.value**
**Low household income***						
Sex	0.02	(-0.04, 0.07)	0.537	0.00	(-0.02, 0.03)	0.761
Childhood SEC	-0.01	(-0.08, 0.07)	0.827	-0.01	(-0.04, 0.03)	0.667
APOE-4	0.05	(-0.02, 0.13)	0.160	-0.01	(-0.04, 0.03)	0.739
**Financial hardships***						
Sex	-0.02	(-0.06, 0.03)	0.483	**0.03**	**(0.01, 0.05)**	**0.008**
Childhood SEC	0.02	(-0.06, 0.09)	0.700	0.01	(-0.03, 0.05)	0.719
APOE-4	-0.03	(-0.08, 0.02)	0.219	0.01	(-0.01, 0.03)	0.258

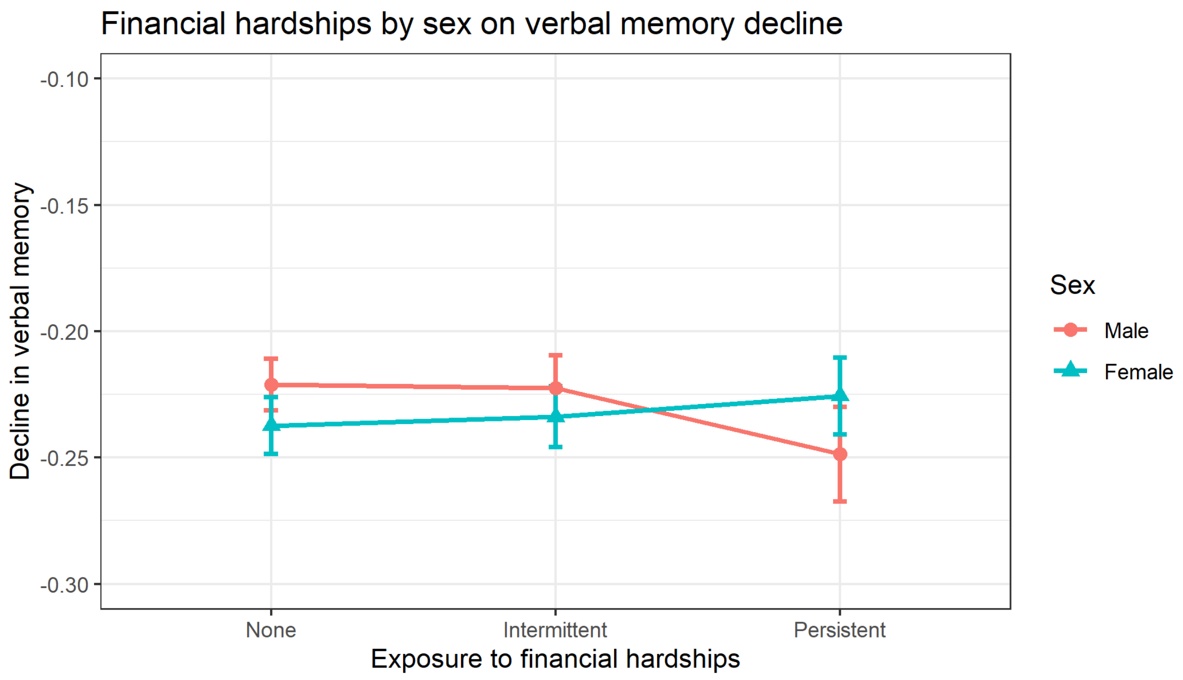

Figure S4. Insight46 sub-sample: Predicted means on verbal memory decline by financial hardships exposure and sex.

3. Repeat primary analyses when low household income is derived within each family size grouping

Table S13. Regression models showing the association between indicators of financial adversity and cognition at endpoint (intercept) and decline over time (slope).

	**Processing speed**						**Verbal memory**					
	**Endpoint (intercept)**			**Decline (slope)**			**Endpoint (intercept)**			**Decline (slope)**		
	**b**	**95%CI**	**p.value**	**b**	**95%CI**	**p.value**	**b**	**95%CI**	**p.value**	**b**	**95%CI**	**p.value**
**Low household income**[a]												
Unadjusted	**-0.10**	**(-0.15, -0.06)**	**0.000**	**0.01**	**(0.00, 0.02)**	**0.022**	**-0.35**	**(-0.40, -0.30)**	**0.000**	**0.01**	**(0.01, 0.02)**	**0.000**
Adjusted	-0.04	(-0.10, 0.01)	0.081	0.01	(-0.00, 0.01)	0.118	**-0.16**	**(-0.20, -0.11)**	**0.000**	**0.00**	**(0.00, 0.01)**	**0.004**
**Financial hardships**												
Unadjusted	**-0.09**	**(-0.14, -0.03)**	**0.001**	0.01	(-0.00, 0.01)	0.101	**-0.23**	**(-0.29, -0.17)**	**0.000**	**0.01**	**(0.01, 0.01)**	**0.000**
Adjusted	-0.05	(-0.10, 0.00)	0.054	0.00	(-0.00, 0.01)	0.223	**-0.10**	**(-0.15, -0.05)**	**0.000**	**0.00**	**(0.00, 0.01)**	**0.020**

[a] Low household income is adjusted for household size

Table S14. Regression models showing the interaction between indicators of financial adversity and sex, childhood SEC, and APOE-4 in relation to cognitive decline, adjusted for all covariates (with low household income adjusted for household size).

	**Processing speed (decline)**			**Verbal memory (decline)**		
	**b**	**95%CI**	**p.value**	**b**	**95%CI**	**p.value**
**Low household income***						
Sex	-0.00	(-0.02, 0.01)	0.646	0.00	(-0.00, 0.01)	0.489
Childhood SEC	-0.00	(-0.03, 0.02)	0.708	0.00	(-0.01, 0.02)	0.400
APOE-4	0.01	(-0.01, 0.02)	0.314	0.00	(-0.01, 0.01)	0.998
**Financial hardships***						
Sex	**-0.01**	**(-0.03, 0.00)**	**0.060**	0.01	(-0.00, 0.01)	0.144
Childhood SEC	0.01	(-0.01, 0.04)	0.259	0.00	(-0.01, 0.01)	0.826
APOE-4	-0.01	(-0.03, 0.01)	0.265	-0.01	(-0.01, 0.00)	0.246

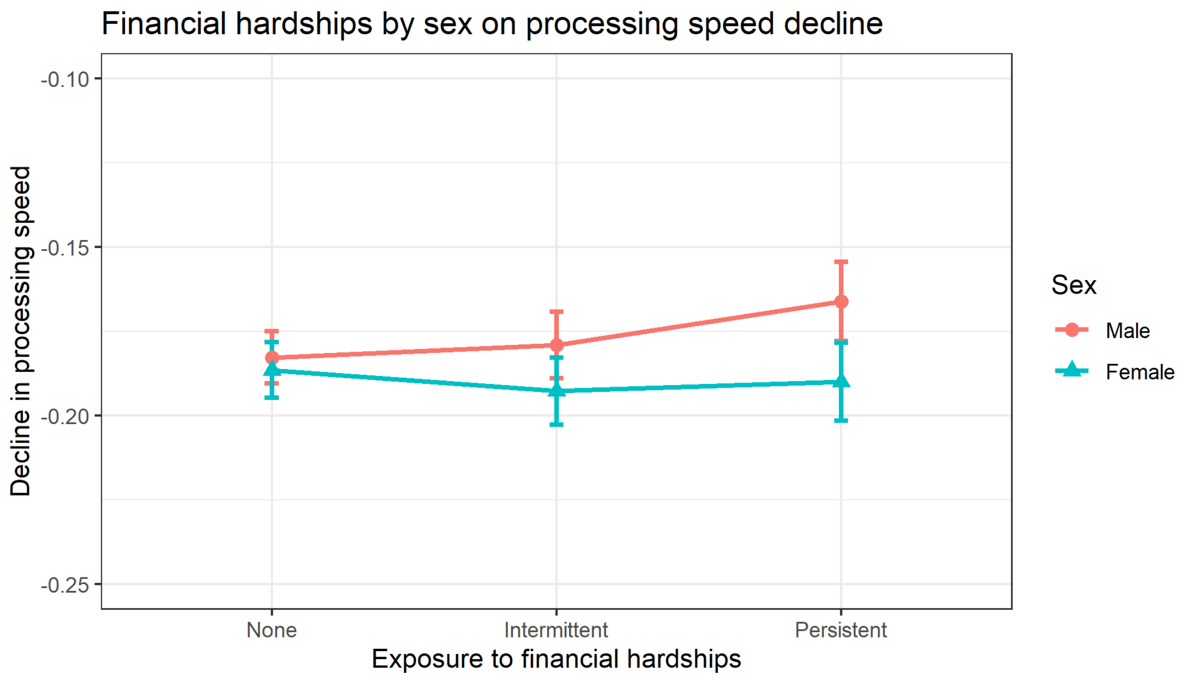

Figure S5. Low household income by household size: Predicted means on processing speed decline by financial hardships exposure and sex.

4. Perform multiple imputation on cognitive scores and estimate cognitive decline and the role of financial adversity within SEM framework

Table S15. Standardised estimates showing the association between financial adversity (low household income and financial hardships) and processing speed and verbal memory (intercept and slope).

	Unadjusted			Adjusted		
	**b**	**95%CI**	**p.value**	**b**	**95%CI**	**p.value**
**Processing speed**						
Intercept ~ low household income	**-0.13**	**(-0.19, -0.07)**	**0.000**	**-0.08**	**(-0.14, -0.02)**	**0.012**
Slope ~ low household income	0.03	(-0.01, 0.06)	0.128	0.03	(-0.01, 0.06)	0.154
Intercept ~ financial hardships	**-0.08**	**(-0.15, -0.02)**	**0.009**	-0.05	(-0.11, 0.01)	0.104
Slope ~ financial hardships	0.01	(-0.03, 0.04)	0.766	0.00	(-0.03, 0.04)	0.857
**Verbal memory**						
Intercept ~ low household income	**-0.30**	**(-0.35, -0.24)**	**0.000**	**-0.13**	**(-0.18, -0.08)**	**0.000**
Slope ~ low household income	0.02	(-0.01, 0.05)	0.269	-0.00	(-0.03, 0.03)	0.886
Intercept ~ financial hardships	**-0.20**	**(-0.26, -0.14)**	**0.000**	**-0.09**	**(-0.14, -0.04)**	**0.001**
Slope ~ financial hardships	0.01	(-0.02, 0.04)	0.579	-0.00	(-0.03, 0.03)	0.801
